# Actinobacterial Diversity in the Sediments of Five Cold Springs on the Qinghai-Tibet Plateau

**DOI:** 10.3389/fmicb.2015.01345

**Published:** 2015-11-30

**Authors:** Jian Yang, Xiaoyan Li, Liuqin Huang, Hongchen Jiang

**Affiliations:** State Key Laboratory of Biogeology and Environmental Geology, China University of GeosciencesWuhan, China

**Keywords:** *Actinobacteria*, diversity, 16S rRNA gene, cold springs, Qinghai-Tibet Plateau

## Abstract

The actinobacterial diversity was investigated in the sediments of five cold springs in Wuli region on the Qinghai-Tibet Plateau using 16S rRNA gene phylogenetic analysis. The actinobacterial communities of the studied cold springs were diverse and the obtained actinobacterial operational taxonomic units were classified into 12 actinobacterial orders (e.g., *Acidimicrobiales, Corynebacteriales, Gaiellales, Geodermatophilales, Jiangellales, Kineosporiales, Micromonosporales, Micrococcales, Nakamurellales, Propionibacteriales, Pseudonocardiales, Streptomycetales*) and unclassified *Actinobacteria*. The actinobacterial composition varied among the investigated cold springs and were significantly correlated (*r* = 0.748, *P* = 0.021) to environmental variables. The actinobacterial communities in the cold springs were more diverse than other cold habitats on the Tibetan Plateau, and their compositions showed unique geographical distribution characteristics. Statistical analyses showed that biogeographical isolation and unique environmental conditions might be major factors influencing actinobacterial distribution among the investigated cold springs.

## Introduction

A large portion of the Qinghai-Tibet Plateau (QTP) is underlain by permafrost, which is suitable for gas hydrate development (Wang and French, 1995; Zhou et al., 2000). Recent evidence indicates that gas hydrate is present in the permafrost zone of Qilian Mountains in the northern margin of QTP (Lu et al., 2009; Zhu et al., 2010). Large numbers of factures and faults are present in the identified hydrate-containing permafrost zone (Lu et al., 2009; Wang, 2010; He et al., 2012), along which cold springs are commonly distributed (Lu et al., 2007; Li et al., 2012).

The environmental condition of the cold springs in the hydrate-containing permafrost zone is similar to marine cold seeps in terms of geochemistry. Cold seeps occur in geologically active and passive continental margins, where continuous methane is advected upward through sediments by forced gradients, supporting abundant microbial populations (Levin, 2005). The methane-fueled communities in marine cold seeps possess high metabolic rates, and they play important roles in carbon and nitrogen cycling (Hinrichs and Boetius, 2002; Boetius and Suess, 2004; Nakagawa et al., 2007; Reeburgh, 2007; Dang et al., 2010). Because of their potentially important role in global climate change, microbial communities in marine cold seeps have received much attention (Sibuet and Olu-Le Roy, 2002; Reeburgh, 2007).

As one of the largest taxonomic units within the *Bacteria* domain, *Actinobacteria* are drawing increasing interests from microbiologists because their biotechnological and commercial value (Goodfellow et al., 1988; Demain, 1995). The characterized actinobacterial strains can be grouped into six known classes: *Acidimicrobiia, Actinobacteria, Coriobacteriia, Nitriliruptoria, Rubrobacteria*, and *Thermoleophilia* (Goodfellow et al., 2012). The actinobacterial diversity and community structures have been investigated in various environments, including marine environments (Goodfellow and Haynes, 1984; Stach et al., 2003; Maldonado et al., 2005; Stach and Bull, 2005; Ward and Bora, 2006), soils (Gremion et al., 2003; Cho et al., 2006; Wu et al., 2009), terrestiral aquatic ecosystems (e.g., freshwater rivers, saline/hypersaline lakes, hot springs, glacial meltwater; Mohagheghi et al., 1986; Mevs et al., 2000; Zwart et al., 2002; Hahn et al., 2003; Warnecke et al., 2004; Mancinelli, 2005; Stach and Bull, 2005; Allgaier and Grossart, 2006; Newton et al., 2007; Hahn, 2009; Holmfeldt et al., 2009; Liu et al., 2009a,b; Song et al., 2009; Wu et al., 2009; Jiang et al., 2010a, 2012a; Ghai et al., 2012, 2014; Goodfellow et al., 2012). These previous studies show that *Actinobacteria* are ubiquitous and actinobacterial community diversity is variable among samples from different ecosystems. The actinobacterial community in marine sediments was mainly composed of the orders of *Acidimicrobiales, Actinomycetales, Corynebacteriales, Frankiales, Micrococcales, Micromonosporales, Pseudonocardiales, Streptomycetales*, and unclassified *Actinobacteria* (Stach et al., 2003; Goodfellow et al., 2012), while the *Actinobacteria* in freshwater ecosystems consisted of acI, acII, acIII, acIV, acSTL, soilII+III, acTH1, and Luna (Hahn et al., 2003; Warnecke et al., 2004; Ghai et al., 2012). In contrast, limited is known about microbial communities in terrestrial cold springs up to date. Previously, one 16S rRNA gene-based microbial study showed the presence of *Actinobacteria* in the cold springs of Wuli, QTP (Li et al., 2012). However, the actinobacterial diversity in these cold springs might be under-represented due to the use of universal bacterial primers (Cottrell and Kirchman, 2000; Jiang et al., 2010a).

The objective of this study was to investigate the actinobacterial diversity and community structure in five Tibetan cold springs based on 16S rRNA gene phylogenetic analyses. We also compared the actinobacterial diversity in the sampled Tibetan cold springs with that in other habitats.

## Materials and Methods

### Site Description and Sample Collection

**FIGURE 1:**
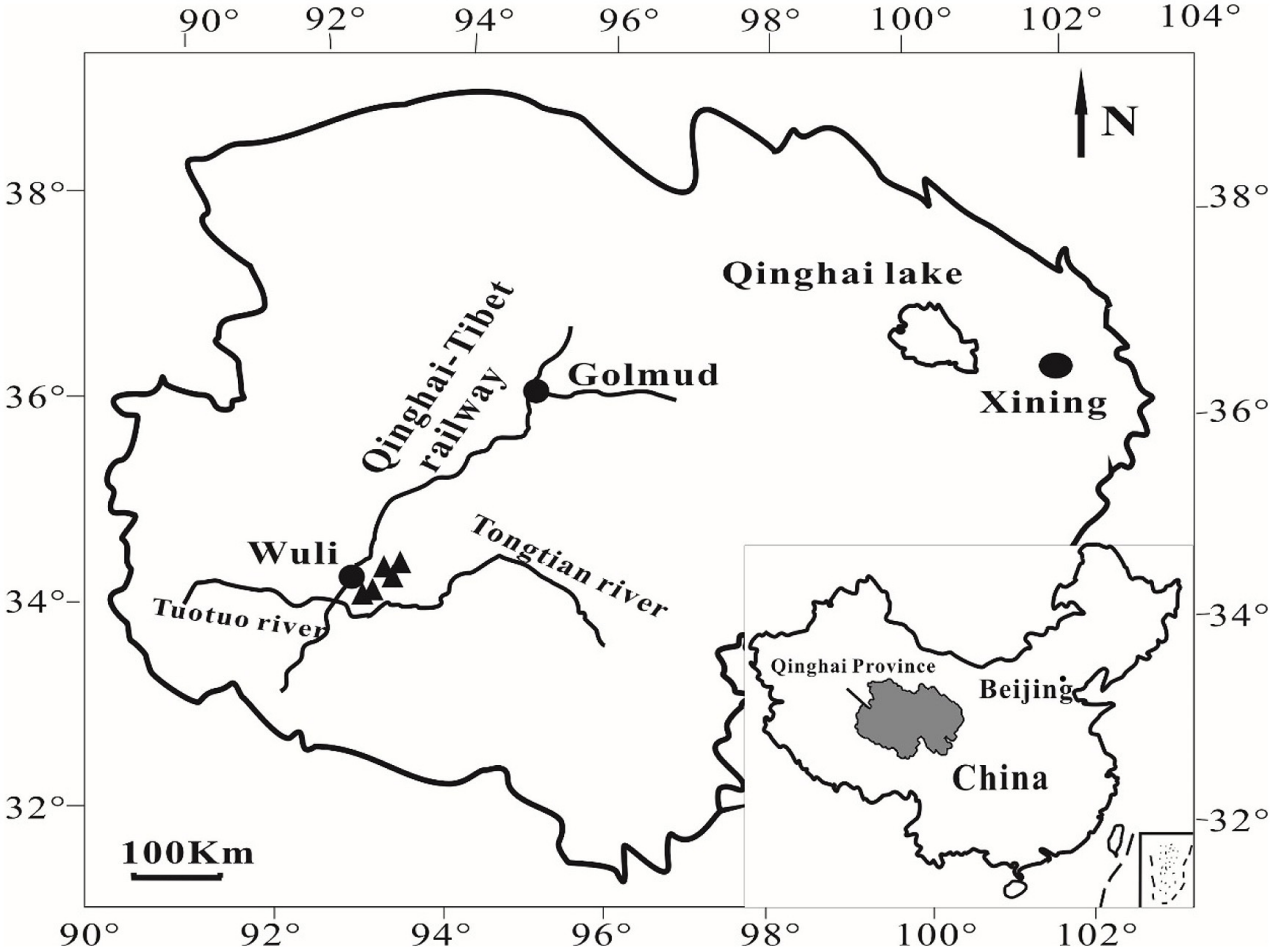
**A geographic map showing the locations of sampling sites in Wuli County, Qinghai-Tibet Plateau, China**.

In July 2010, five cold springs were sampled in Wuli Area (**Figure 1**), Qinghai Province, China, that is adjacent to the Daha coal mine (Zhou, 2004) and located in the Fenghuo Mountain-Wuli gas hydrate zone (Zhu et al., 2011). The Wuli area is located at the elevation of ∼4600 m. Water pH and temperature were measured in the field using a digital soil pH meter (Ferrymorse-Seed Company) and a mercury thermometer, respectively. During sample collection (around noon), the ambient temperature was 15–17°C, whereas the water temperature of the sampled cold springs was around 1–3°C. Sediments from five cold springs (named as QCS1, QCS3, QCS4, QCS5, and QCS6, respectively) were collected into 50 mL sterile Falcon tubes using a sterile spatula. The collected samples were stored at -20°C in the field as well as during transportation and subsequently at -80°C in the laboratory until further analyses.

### Porewater Chemistry and Sediment Mineralogy

Cation composition of pore water was analyzed by using inductively coupled plasma-optical emission spectrometry (ICP-OES; Varian Vista MPX, Varian, Palo Alto, CA, USA). Anion composition was analyzed using ionic chromatography (IC) on a Dionex ISC90 equipped with a conductivity detector and an AS14A column (eluent, 10 μM Na_2_CO_3_/NaHCO_3_; flow rate, 1.0 mL/min; Jiang et al., 2010a). The sediment mineralogy was analyzed by using powder X-ray diffraction (XRD) on a Rigaku D/Max 2550/PC X-ray diffractometer with Cu Ka radiation (40 kV; 100 mA; Zhang et al., 2009).

### DNA Extraction, PCR, and Phylogenetic Analyses

DNA of the sediment samples was extracted using FastDNA^®^ SPIN Kit for Soil (MP Biomedicals, LLC, Solon, OH, USA) according to the manufacturer’s protocols. The actinobacterial 16S rRNA gene from the extracted DNA samples was amplified using the actinobacterial 16S rRNA gene-specific forward primer S-C-Act-0235-a-S-20 (5′-CGC GGC CTA TCA GCT TGT TG-3′) and reverse primer S-C-Act-0878-a-A-19 (5′-CCG TAC TCC CCA GGC GGG G-3′; Stach et al., 2003) with the same PCR conditions as described previously (Wu et al., 2009). PCR products were purified using Agarose Gel DNA Fragment Recovery Kit Ver. 2.0 (TaKaRa, Dalian, China) according to the manufacturer’s instructions. 16S rRNA gene clone libraries were constructed by ligating the purified PCR products into pGEM^®^-T Easy Vector system (Promega, Madison, WI, USA) and transformed into competent *Escherichia coli* JM109 cells according to the manufacturer’s protocols. Positive clones were randomly picked for sequencing with an ABI 3730 XL DNA Sequencer (Applied BioSystems, Foster City, CA, USA). Rarefaction analysis was performed to evaluate the saturation of the sampled clones using the PAST software package^1^ (see Supplementary Figure S1).

All the obtained clone sequences were assembled and edited by using Sequencher v.4.1 (GeneCodes, Ann Arbor, MI, USA) and then checked by BLAST function in NCBI (National Center of Biotechnology Information^2^). Potential chimeric sequences were removed from further analyses. Operational taxonomic units (OTUs) were identified at a 97% cutoff by using Mothur v1.36.1 with furthest neighbor method (Schloss et al., 2009). One sequence from each OTU was selected and the closest references were picked up from the GenBank database for phylogenetic analyses (see Supplementary Table S1). The representative sequences of OTUs and references were combined and aligned using ClustalW in MEGA (molecular evolutionary genetics analysis) program, version 6.06. Maximum likelihood phylogenetic trees were constructed using the above aligned sequences. Bootstrap replications of 1000 were assessed. The unique clone sequences determined in this study were deposited in the GenBank database under accession numbers JX667788–JX667977, JF712624–JF712648, and KU052203–KU052216.

### Statistical Analysis

Alpha-diversity indices, such as Simpson, Shannon, Equitability and Chao 1, were calculated by using the PAST software package (Hammer et al., 2001). Coverage values of the clone libraries were calculated with the equation *C* = 1-*n*/*N*, where *n* was the number of phylotypes that occurred only once in the clone library and *N* was the total number of sequenced clones (Jiang et al., 2010b). All obtained environmental variables were normalized (values ranged between 1 and 100) to improve normality and homoscedasticity for statistical analyses. Clustering analysis were performed by using PAST software package with unweighted pair group method with arithmatic mean. Mantel tests were performed to assess the correlation between actinobacterial community composition and environmental variables by using the PAST software package. Briefly, the biotic matrices were constructed on the basis of Bray-Curtis dissimilarity of actinobacterial community compositions. The abiotic matrices were constructed on the basis of the Euclidean distances of normalized environmental variables.

In order to compare the actinobacterial community composition difference between the QTP cold springs and other related habitats, reference actinobacterial clone sequences from Tibetan hot springs (Jiang et al., 2012a), Tibetan (hyper-)saline lakes (Jiang et al., 2010a), freshwater sample of Daotang river (Jiang et al., 2010a), Atlantic ocean deep-sea sediment (Stach et al., 2003), the Three Gorges Dam of the Yangtze River (Jiang et al., 2012b) and Tengchong hot springs (Song et al., 2009) were downloaded from the GenBank database and combined with the ones obtained in this study. In order to avoid any bias resulting from different primers, only actinobacterial 16S rRNA sequences amplified from the same primer set and PCR protocol as this study were included in subsequent analysis. The combined actinobacterial 16S rRNA sequences were aligned using ClustalW in MEGA and then were subjected to OTU identification at the 97% cutoff using Mothur v1.36.1 with furthest neighbor method (Schloss et al., 2009). Clustering analysis was performed to discern the difference of actinobacterial community composition among habitats based on Jaccard similarity using the PAST software package.

## Results

### Porewater Chemistry and Mineralogy

The pH of the sampled cold springs were neutral, and the temperature ranged 1.5–2.5° (**Table 1**). The concentration of Si^4+^ and total Fe were 0.6–5.1 and 0.0–6.9 mg/L, respectively. Heavy metals Mn and Sr only occurred in the QCS1 sample. The sediment samples were mainly composed of quartz, plagioclase, calcite, montmorillonite, illite, and kaolinite.

**Table 1:** Geographic and geochemical parameters of the studied cold springs on the Qinghai-Tibet Plateau.

Sample ID	QCS1	QCS3	QCS4	QCS5	QCS6
GPS location (N/E)	34°20′/ 94°38′	34°20′36.7″/ 92°44′51.6″	34°20′42.5″/ 92°45′1.5″	34°20′53.8″/ 92°45′29.3″	34°21′19.7″/ 92°45′29.7″
Elevation (m)	4610	4611	4609	4637	4612
Temperature (°C)	2.5	1.5	2.5	2	2
pH	7	6.8	7	7.2	7
Mg^2+^ (mg/L)	40.8	57.9	6	41	56.7
Si^4+^ (mg/L)	2.7	3.2	0.6	3.5	5.1
Ca^2+^ (mg/L)	50.1	84.2	12.7	90.4	185.2
K^+^ (mg/L)	5.19	5.4	4.6	9.2	96
Na^+^ (mg/L)	104.2	97.5	8.7	55.8	69.1
F^-^ (mg/L)	0	4.8	2	5.1	13
Cl^-^ (mg/L)	133	883.1	75.1	390.7	372
NO_3_^-^ (mg/L)	3.6	98.3	40.4	165.5	26
PO_4_^2-^ (mg/L)	0.4	0.0	0.0	0.0	0.0
SO_4_^2-^ (mg/L)	159.1	717.5	146.2	1609.2	3057
Total Fe (mg/L)	0.0	0.2	1.8	0.1	6.9
Total Mn (mg/L)	0.1	0.0	0.0	0.0	0.0
Total Sr (mg/L)	1.0	0.0	0.0	0.0	0.0

### Phylogenetic Diversity of *Actinobacteria*

**FIGURE 2:**
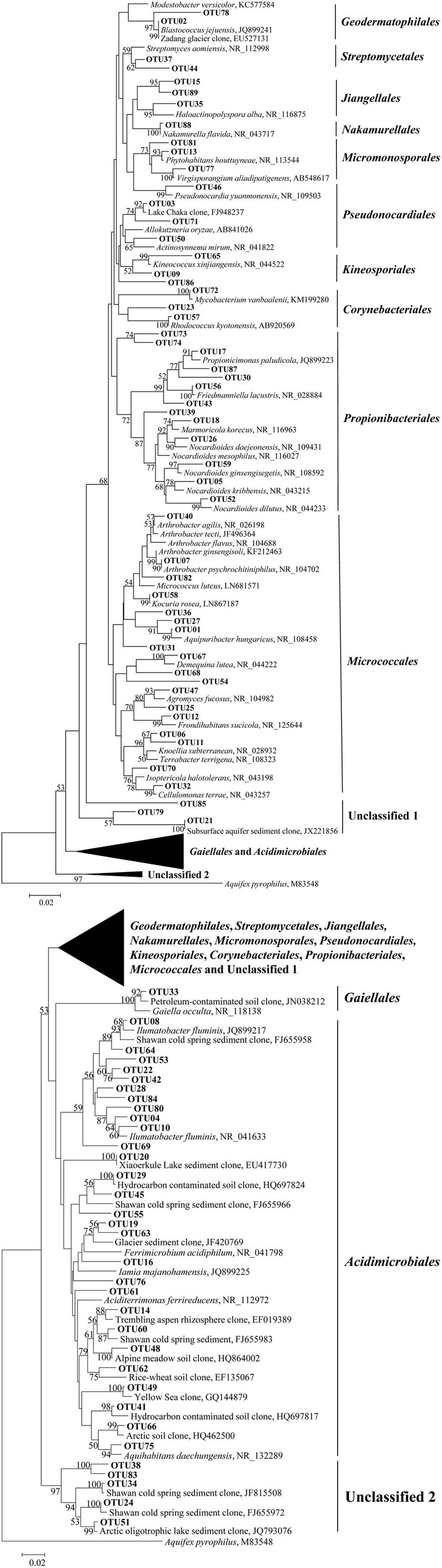
**ContinuedMaximum-likelihood tree (partial sequences, ∼640 bp) showing the phylogenetic relationships of the actinobacterial 16S rRNA gene sequences cloned from the studied samples to closely related sequences from the GenBank database.** One representative clone sequence within each OTU was shown. Bootstrap values of >50% (for 1000 iterations) were shown.

**FIGURE 3:**
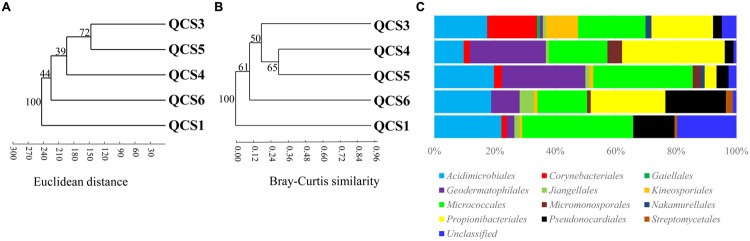
**(A)** Cluster analysis of environmental variables in the studied samples based on Euclidean distance; **(B)** Cluster analysis of actinobacterial community composition in the studied samples based on Bray-Curtis similarity; **(C)** Schematic figures showing the frequencies of OTUs affiliated with major actinobacerial orders in this study.

Five clone libraries (QCS1, QCS3, QCS4, QCS5, and QCS6) were constructed. A total of 484 actinobacterial 16S rRNA gene clone sequences were obtained: 117, 85, 76, 103, and 103 clone sequences for QCS1, QCS3, QCS4, QCS5, and QCS6, respectively. The number of sequenced clones represented 76–91% coverage for each clone library (**Table 2**). Out of these clone sequences, one hundred and twenty OTUs (29, 27, 32, 27, 31 for QCS1, QCS3, QCS4, QCS5, and QCS6, respectively) were identified (**Table 2**). These identified OTUs could be classified into *Acidimicrobiales, Corynebacteriales, Gaiellales, Geodermatophilales, Jiangellales, Kineosporiales, Micromonosporales, Micrococcales, Nakamurellales, Propionibacteriales, Pseudonocardiales, Streptomycetales*, and unclassified *Actinobacteria* (**Figure 2**). The diversity indices such as Shannon (2.6–3.0), Chao 1 (34.3–46.2) varied among the studied cold springs (**Table 2**). *Acidimicrobiales, Geodermatophilales, Micrococcales, Propionibacteriales*, and *Pseudonocardiales* were dominant actinobacterial groups (**Figure 3C**). Among the studied samples, *Acidimicrobiales, Micrococcales, Pseudonocardiales*, and unclassified *Actinobacteria* were dominant (relative abundance > 10%) in the QCS1 sample; *Acidimicrobiales, Micrococcales, Pseudonocardiales*, and *Propionibacteriales* dominated in the QCS3 sample; *Acidimicrobiales, Geodermatophilales, Micrococcales*, and *Propionibacteriales* were dominant in the QCS4 and QCS5 samples; and *Acidimicrobiales, Corynebacteriales, Kineosporiales, Micrococcales*, and *Propionibacteriales* dominated in the QCS6 sample (**Figure 3C**).

**Table 2:** Ecological estimates and major group affiliation of clone sequences retrieved from the five cold springs on the Qinghai-Tibet Plateau.

Clone libraries	QCS1	QCS3	QCS4	QCS5	QCS6
Library sizes (No. of clones)	117	85	76	103	103
Coverage (%)	91	85	76	88	86
No. of observed OTUs	29	27	32	27	31
Simpson	0.9	0.9	0.9	0.9	0.9
Shannon	2.9	2.8	2.9	2.6	3.0
Equitability	0.9	0.8	0.8	0.8	0.9
Chao 1	34.6	36.8	47.3	34.3	46.2

The order of *Micrococcales* was the most dominant (average abundance 25.6%) group in the studied cold spring samples, and a large portion of clones affiliated with *Micrococcales* were closely related (identity: 95–99%) to cultured psychrophilic *Actinobacteria*, such as *Arthrobacter* sp. (Reddy et al., 2000; Fong et al., 2001; Wang et al., 2009) and *Demequina* sp. (Finster et al., 2009; **Figure 2** and Supplementary Table S1). Furthermore, many clone sequences obtained in this study were affiliated with *Acidimicrobiales*, and they were related to clone sequences retrieved from cold habitats such as arctic soil exposed by glacier retreat (Quince et al., 2011), cold spring sediment in Shawan, Xinjiang, China (Zeng et al., 2010), and Shule River permafrost soils on the Tibetan Plateau (**Figure 2**). The remaining 5.9% (32 out of 484) of the clone sequences retrieved in this study belonged to unclassified *Actinobacteria* (**Figure 2**).

### Relationships between Actinobacterial Community Composition and Environmental Variables

**FIGURE 4:**
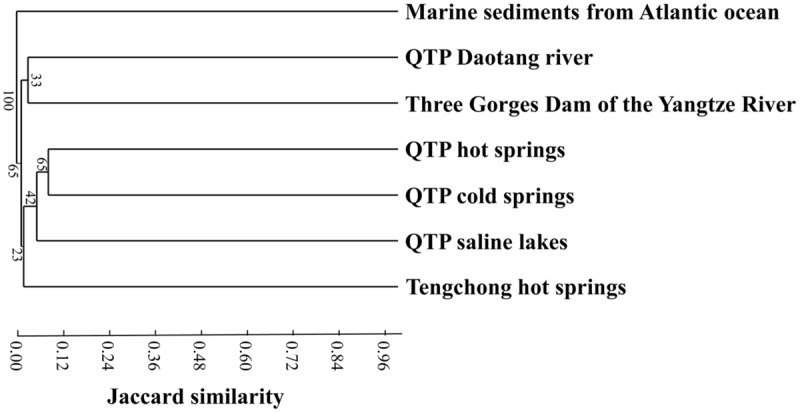
**Jaccard similarity-based cluster analysis showing the differences between actinobacterial 16S rRNA gene clone libraries of the QTP cold springs in this study and those from hot springs on the QTP (Jiang et al., 2012a), (hyper-)saline lakes on the QTP (Jiang et al., 2010a), freshwater sample of Daotang river on the QTP (Jiang et al., 2012b), Tengchong hot springs of Yunnan Province, China (Song et al., 2009), Atlantic ocean deep-sea sediment in the edge of the Saharan debris flow near the Canary Islands (Stach et al., 2003), and waters near the Three Gorges Dam in the middle reach of the Yangtze River (Jiang et al., 2012b)**.

Cluster analysis showed that the cold spring geochemistry (**Figure 3A**) presented similar grouping patterns to actinobacterial community composition (**Figure 3B**) among the studied samples. Mantel tests showed that actinobacterial community composition of the studied cold springs was significantly correlated (*r* = 0.748, *P* = 0.021) with the combined environmental variables but not significantly (*P* > 0.05) with any single environmental variable measured in this study. Furthermore, cluster analysis showed that the actinobacterial communities in the QTP samples (including clod springs, hot springs and lakes) were grouped into one cluster, which has little similarity (Jaccard similarity < 0.05) with that of marine sediments from Atlantic ocean and Tengchong hot springs (**Figure 4**).

## Discussion

### Actinobacterial Communities in the QTP Cold Springs

The actinobacterial community composition in cold springs on the QTP was similar to that of cold habitats in other locations. The actinobacterial communities of the studied QTP cold springs were composed of major groups related to psychrophilic *Actinobacteria* species (e.g., *Arthrobacter psychrochitiniphilus, Demequina lutea*) and environmental clone sequences retrieved from cold habitats, such as snow/ice and soils in Qinghai–Tibetan Plateau and Arctic/Antarctic. This indicated that low temperature was a major environmental factor for dominating actinobacterial distribution in cold habitats.

Excluding low-temperature property, actinobacterial community composition in the studied cold springs may be affected by environmental variable composition. For example, samples of QCS3, QCS4, and QCS5 had similar environmental variables composition, and thus possessed similar actinobacterial community compositions (**Figures 3A,B**); the environmental variable composition of QCS1 and QCS6 was different from the other studied samples (**Figure 3A**): QCS1 possess highest concentration of Na^+^ and heavy metal Mn and Sr (**Table 1**), and QCS6 sample has highest Ca^2+^ and total Fe (**Table 1**), thus it is reasonable to observe distinct actinobacterial community compositions in QCS1 and QCS6 samples from that in QCS3, QCS4, and QCS5 samples (**Figure 3B**). Previous studies have shown that microbial community composition could be affected by multiple environmental parameters, such as salinity (Lozupone and Knight, 2007), temperature (Lindh et al., 2013), and heavy metals (Gong et al., 2015). Therefore, it is not surprising to observe significant correlation between actinobacterial community composition and environmental variables in the studied cold springs.

It is notable that some of the retrieved actinobacterial clone sequences from the cold springs showed high identity with those obtained from petroleum- or coal-related environments. This observation is expected in that the sampling sites in this study was located in the Wuli-Daha coal-bearing belt (Zhou, 2004) and Fenghuo Mountain-Wuli gas hydrate-bearing belt (Zhu et al., 2010) in southern Qinghai Province. The underlying coal or gas hydrate might provide abundant nutrients, which support diverse actinobacterial communities in the studied cold springs (Santos et al., 2008; Jiang et al., 2010a).

### Actinobacterial Difference between the QTP Cold Springs and Other Habitats

The actinobacterial community in the investigated cold springs was more diverse than other cold environments. For example, the *Actinobacteria* sequences obtained in this study were distributed into 12 orders (**Figures 2** and **3C**). In contrast, the *Actinobacteria*-related clones retrieved in the snow of four glaciers on the Tibetan Plateau were mainly affiliated with the order *Micrococcales* and unclassified *Actinobacteria* (Liu et al., 2009b). This suggested Tibetan cold springs might contain more suitable growth conditions for *Actinobacteria* than glaciers.

Actinobacterial communities from different habitats possessed certain geographic characteristics. The actinobacterial clones from the studied cold springs (this study) were closely related to those from the QTP hot springs and saline lakes (**Figure 4**), this indicated that the actinobacterial communities in the studied cold springs were more similar to that in other QTP samples (including hot springs and lakes) than to those in the samples from other locations. For example, the majority of the retrieved actinobacterial 16S rRNA gene clone sequences in the investigated cold springs were affiliated with *Micrococcales, Propionibacteriales*, and *Acidimicrobiales*. Actinobacterial clones retrieved from Tibetan saline lakes were mainly classified with *Micrococcales, Propionibacteriales*, and *Frankiales* (Jiang et al., 2010a). In contrast, the actinobacterial communities in Tengchong hot springs were mainly affiliated with unclassified *Actinobacteria, Rubrobacterales*, and *Frankiales* (Song et al., 2009). Previous studies have shown that *Actinobacteria* in hot springs, soils and oceans possess geographic distributions (Ward and Bora, 2006; Wawrik et al., 2007; Valverde et al., 2012). In addition, the *Actinobacteria* communities in the studied QTP cold spring sediments were different from those in marine sediments (Stach et al., 2003; Goodfellow et al., 2012) and freshwater ecosystems (Hahn et al., 2003; Warnecke et al., 2004; Ghai et al., 2012). The observed geographic distribution of *Actinobacteria* in the QTP samples could be ascribed to the distinct conditions (e.g., dry climate, low pressure, high intensity of UV radiation) of the cold springs, hot springs, and saline lakes on the QTP from other ecosystems (Jiang et al., 2010a, 2012a). However, the underlying reasons still await further investigation.

In summary, the actinobacterial communities in the studied Tibetan cold springs possessed unique compositional characteristics and were mainly consisted of *Acidimicrobiales, Corynebacteriales, Gaiellales, Geodermatophilales, Jiangellales, Kineosporiales, Micromonosporales, Micrococcales, Nakamurellales, Propionibacteriales, Pseudonocardiales, Streptomycetales*, and unclassified *Actinobacteria*. Biogeographical isolation and unique environmental conditions might be predominant factors affecting the observed similarities and differences in the actinobacterial communities between the investigated cold springs and other habitats.

## Conflict of Interest Statement

The authors declare that the research was conducted in the absence of any commercial or financial relationships that could be construed as a potential conflict of interest.

## Footnotes

1 http://folk.uio.no/ohammer/past/

2 http://blast.ncbi.nlm.nih.gov/Blast.cgi
