# Supplementary material for: Actinobacterial Diversity in the Sediments of Five Cold Springs on the Qinghai-Tibet Plateau
Source: Front Microbiol. 2015 Nov 30;6:1345. doi: 10.3389/fmicb.2015.01345 (PMC4663260; doi:10.3389/fmicb.2015.01345)
Supplement: Supplementary file 2 [file Data_Sheet_2.DOCX]

Figure caption:

Fig. S1 Rarefaction curves of the clone libraries in this study.

Fig. S1
